# Association of TREM-1, IL-1β, IL-33/ST2, and TLR Expressions With the Pathogenesis of Ocular Toxoplasmosis in Mouse Models on Different Genetic Backgrounds

**DOI:** 10.3389/fmicb.2019.02264

**Published:** 2019-10-09

**Authors:** Yanxia Zhang, Jian He, Huanqin Zheng, Shiguang Huang, Fangli Lu

**Affiliations:** ^1^Department of Parasitology, Zhongshan School of Medicine, Sun Yat-sen University, Guangzhou, China; ^2^Key Laboratory of Tropical Disease Control of Ministry of Education, Sun Yat-sen University, Guangzhou, China; ^3^Public Experimental Teaching Center, Sun Yat-sen University, Guangzhou, China; ^4^School of Stomatology, Jinan University, Guangzhou, China

**Keywords:** ocular toxoplasmosis, C57BL/6 mice, BALB/c mice, TREM-1, IL-1β, IL-33/ST2, TLRs, neutrophils

## Abstract

Ocular toxoplasmosis (OT) is one of the most common causes of posterior uveitis. The signaling of triggering receptor expressed on myeloid cells (TREM)-1 amplifies inflammation, whereas TREM-2 signaling is anti-inflammatory. IL-1β is a major driver of inflammation during infection. Toll-like receptors (TLRs) play important roles in protective immune response during *Toxoplasma gondii* infection, and interleukin (IL)-33 receptor (T1/ST2) signaling prevents toxoplasmic encephalitis in mice. However, the pathogenic mechanisms of OT are not yet well elucidated. To investigate the role of TREM-1, TREM-2, IL-1β, IL-33/ST2, and TLRs in OT of susceptible C57BL/6 (B6) and resistant BALB/c mice, both strains of mice were intravitreally infected with 500 tachyzoites of the RH strain of *T. gondii*. Histopathological analysis showed that *T. gondii*-infected B6 mice had more severe ocular damage observed by light microscopy, higher number of neutrophil elastase-positive cells in the eyes detected by immunohistochemical staining, more *T. gondii* tachyzoites in the eyes observed by transmission electron microscopy, and higher mRNA expression levels of tachyzoite-specific surface antigen 1 detected by quantitative real-time reverse transcription-polymerase chain reaction (qRT-PCR) in comparison of *T. gondii*-infected BALB/c mice. Detected by using qRT-PCR, the mRNA expression levels of TREM-1, IL-1β, IL-33, ST2, TLR11, TLR12, and TLR13 were significantly higher in the eyes of *T. gondii-*infected B6 mice than those of *T. gondii-*infected BALB/c mice, whereas the mRNA expression levels of TLR3 and TLR9 were significantly higher in the eyes of *T. gondii-*infected BALB/c mice than those of *T. gondii-*infected B6 mice. Correlation analysis showed that significant positive correlations existed between TREM-1 and IL-1β/IL-33/ST2/TLR9/TLR11 in the eyes of B6 mice and existed between TREM-1 and IL-33/ST2/TLR3/TLR9/TLR13 in the eyes of BALB/c mice after ocular *T. gondii* infection. Our data revealed that, compared with *T. gondii*-resistant BALB/c mice, ocular *T. gondii* infection can stimulate higher production of TREM-1, IL-33, ST2, TLR11, TLR12, and TLR13 in the eyes of *T. gondii*-susceptible B6 mice, however, whether those lead to more severe ocular pathology in the susceptible B6 mice remain to be further studied.

## Introduction

Ocular toxoplasmosis (OT) is caused by *Toxoplasma gondii* infection, with potentially vision-threatening complications such as retinal detachment, choroidal neovascularization, and glaucoma (Park and Nam, 2013; Garweg, 2016), which involves typically the posterior part of eye but results in different clinical symptoms based on the involved area and level of inflammation (Ali-Heydari et al., 2013; Laboudi and Sadak, 2017). Eye injuries caused by *T. gondii* infection affect the retina and the choroid with local inflammatory reactions (Maenz et al., 2014). Acquired infections may account for a larger portion of OT than congenital toxoplasmosis (Atmaca et al., 2004).

It has been reported that genetic factors are major determinants for susceptibility to infection with *T. gondii* (Deckert-Schluter et al., 1994). Our previous study found that compared with both BALB/c and CBA/J mice, ocular infection of C57BL/6 (B6) mice with *T. gondii* resulted in severe inflammatory lesions and high numbers of parasites in eye tissue, and higher serum levels of gamma interferon and tumor necrosis factor alpha (TNFα), indicating that genetic factors of the host are critical in determining susceptibility to experimental OT in murine models (Lu et al., 2005). Our recent study demonstrated that B6 mice expressed higher levels of Gal-9 and its receptors (Tim-3 and CD137) in the eye tissues than those in BALB/c mice following ocular *T. gondii* infection (Chen et al., 2017). However, so far genetic factors in the pathogenesis and course of OT still remain unclear.

The triggering receptor expressed on myeloid cells (TREM) family including TREM-1, TREM-2, TREM-3, and TREM-4 have been identified, in which TREM-1 activation amplifies inflammation, whereas TREM-2 activation is anti-inflammatory (Klesney-Tait et al., 2006; Turnbull et al., 2006; Watarai et al., 2008). TREM-1 can trigger the release of proinflammatory cytokines such as interleukin (IL)-1β, IL-6, and TNFα; crucially amplify both acute inflammatory responses and chronic inflammation (Bouchon et al., 2000; Bleharski et al., 2003; Schenk et al., 2007). TREM-1 works synergistically with toll-like receptors (TLRs) and Nod-like receptors to increase proinflammatory reactions (Bouchon et al., 2001; Netea et al., 2006). In addition, TREM-1 plays critical roles in fungal keratitis and increases with growing keratomycosis severity (Hu et al., 2014). TREM-2 promotes host resistance to *Pseudomonas aeruginosa* infection by suppressing corneal inflammation (Sun et al., 2013).

IL-1β is an important inflammation mediator and a proinflammatory cytokine (Zhang et al., 2018), which is involved in multiple cellular activities such as cell proliferation, apoptosis, and differentiation (Zhao et al., 2018). IL-33 is a member of the IL-1 family and has been identified as a mediator of various inflammatory diseases such as asthma, cardiovascular diseases, and allergic diseases (Liew et al., 2016). ST2 is defined as the IL-33 receptor (Carriere et al., 2007). It has been reported that ST2/IL-33 signaling implicates in protection from various infections (Griesenauer and Paczesny, 2017). When IL-33 receptor (T1/ST2)-deficient BALB/c mice were infected with *T. gondii*, they showed increased pathology and increased parasite transcript levels in the brain, indicating T1/ST2 signaling is necessary to prevent the development of toxoplasmic encephalitis (Jones et al., 2010).

Toll-like receptors play an important role in initiating immune responses against many pathogens, including *T. gondii*. TLR/MyD88 signaling pathway is the key pathway in initiating defense against *T. gondii* (Denkers, 2010). TLR2 and TLR4 contribute to the recognition and stimulation of immunity to *T. gondii* and participate in the host protection to *T. gondii* infection (Mukherjee et al., 2016). TLR3 induces type I interferon responses via parasite RNA (Beiting et al., 2014). TLR4 and TLR9 single nucleotide polymorphisms are involved in protection against congenital toxoplasmosis (Wujcicka et al., 2015). Mice lacking TLR9 significantly reduce intestinal pathology, lose weight, and live longer than wild-type mice (Minns et al., 2006). A study has proved a role for TLR9 in initiating proinflammatory responses that cause severe OT in Brazil (Peixoto-Rangel et al., 2009). TLR11 can interact with *T. gondii* profilin-like protein to elicit immune responses (Hatai et al., 2016). TLR12 can function alone in plasmacytoid dendritic cells and interact with TLR11 to specifically recognize and respond to *T. gondii* profilin (Koblansky et al., 2013). Quadruple TLR3/TLR7/TLR9/TLR11 deficient mice showed diminished resistance to *T. gondii* infection, indicating they play an essential role in toxoplasmosis (Andrade et al., 2013). So far, the role of TREMs and TLRs in OT remains poorly understood. Therefore, this study intended to investigate the expression and role of TREM-1, TREM-2, IL-1β, IL-33, ST2, and TLRs in OT in mouse models on different genetic backgrounds.

## Materials and Methods

### Mice, *T. gondii* Parasites, and Intravitreal Infection

Female B6 and BALB/c mice, 8–10 weeks old, were purchased from the animal facility at Sun Yat-sen University in Guangzhou, China. *T. gondii* RH strain tachyzoites were maintained in Vero or human foreskin fibroblast cells grown in Dulbecco’s modified Eagle medium (Invitrogen, Carlsbad, CA, United States) supplemented with 5% fetal bovine serum at 37°C with 5% CO_2_.

Mice were injected into the mid-vitreous with 1 μL parasite suspension in sterile phosphate-buffered saline (PBS, pH 7.4) containing 500 tachyzoites or the same volume of PBS using a 10-μL Hamilton microsyringe (Charles et al., 2007). A total of 24 mice of each strain were used in the experiments: 12 mice of each strain were intraocularly injected with 500 tachyzoites of *T. gondii* and 12 mice of each strain were injected with equal volume of PBS as negative controls. Mice were euthanized at day 8 post infection (p.i.), and the eyes were enucleated for further analysis.

### mRNA Expression Analysis by Using Quantitative Real-Time Reverse Transcription-Polymerase Chain Reaction (qRT-PCR)

Total RNA was extracted from the eyes of each mouse using a RNA Extraction Kit (TaKaRa Bio Inc., Shiga, Japan) according to the manufacturer’s protocol. The absorbance values at 260 and 280 nm were used to estimate total RNA purity (NanoDrop Technologies, DE, United States). For cDNA synthesis, a PrimeScript^TM^ II 1st Strand cDNA Synthesis Kit (TaKaRa Bio Inc.) was used; total amount of RNA used in qRT-PCR for each sample was 1 μg. To determine tissue mRNA levels of TREM-1, TREM-2, IL-1β, IL-33, ST2, TLR2, TLR3, TLR4, TLR5, TLR7, TLR9, TLR11, TLR12, and TLR13, qRT-PCR was performed using SYBR Green qPCR Master Mix (TaKaRa Bio Inc.) following the manufacturer’s instructions. For eye parasite burden, mRNA level of tachyzoite-specific surface antigen 1 (SAG1) was measured by qRT-PCR as previously described (Liu et al., 2018). All qRT-PCR reactions were performed in duplicate. Primers used for qRT-PCR are listed in Table 1. The results are expressed as relative mRNA levels whereby the expression in naive mice was arbitrarily set at 1 (van der Kraan et al., 2001). Relative mRNA expressions of each target gene were normalized to that of the housekeeping gene, β-actin, and the results are expressed as fold change compared with uninfected controls.

**TABLE 1:** Primer sequences of mouse target cytokines and housekeeping gene used for quantitative real-time polymerase chain reaction assays.

**Genes**	**Primer sequence (5′→3′)**	**Accession**
SAG1	Forward primer ATGTCGCTTCTTAGCCGAGT	XM_002365028.1
	Reverse primer TCACAGGAAGTTGCTTCAGG	
β-actin	Forward primer TGGAATCCTGTGGCATCCATGAAAC	NM_007393.5
	Reverse primer TAAAACGCAGCTCAGTAACAGTCCG	
TLR2	Forward primer GCAAACGCTGTTCTGCTCAG	NM_011905.3
	Reverse primer AGGCGTCTCCCTCTATTGTATT	
TLR3	Forward primer GTGAGATACAACGTAGCTGACTG	NM_126166.5
	Reverse primer TCCTGCATCCAAGATAGCAAGT	
TLR4	Forward primer ATGGCATGGCTTACACCACC	NM_021297.3
	Reverse primer GAGGCCAATTTTGTCTCCACA	
TLR5	Forward primer TGGGGACCCAGTATGCTAACT	NM_016928.3
	Reverse primer CCACAGGAAAACAGCCGAAGT	
TLR7	Forward primer ATGTGGACACGGAAGAGACAA	NM_133211.4
	Reverse primer ACCATCGAAACCCAAAGACTC	
TLR9	Forward primer ATGGTTCTCCGTCGAAGGACT	NM_031178.2
	Reverse primer GAGGCTTCAGCTCACAGGG	
TLR11	Forward primer TCCCTGATTGCATCATAGCAGA	NM_205819.3
	Reverse primer GGGCCGAGGTACAGAATGG	
TLR12	Forward primer CCTGGTCTCCCGCTATTTCAC	NM_205823.2
	Reverse primer CCGAGGTACAACTTCCAAGGT	
TLR13	Forward primer GTTGTAACCTGGATGCCTAAGAC	NM_205820.1
	Reverse primer GGCCTCTGTCAAGTTGGTGA	
TREM-1	Forward primer CCTGTTGTGCTCTTCCATCCTG	NC_000083.6
	Reverse primer CGGGTTGTAGTTGTGTCACTGG	
TREM-2	Forward primer CTACCAGTGTCAGAGTCTCCGA	NC_000083.6
	Reverse primer CCTCGAAACTCGATGACTCCTC	
ST2	Forward primer CAAGTAGGACCTGTGTGCCC	NC_000067.6
	Reverse primer CGTGTCCAACAATTGACCTG	
IL-33	Forward primer TCCAACTCCAAGATTTCCCCG	NC_000085.6
	Reverse primer CATGCAGTAGACATGGCAGAA	
IL-1β	Forward primer CGCAGCAGCACATCAACAAGAGC	NC_000068.7
	Reverse primer TGTCCTCATCCTGGAAGGTCCACG	

### Histopathology

Mice were euthanized at day 8 p.i., and the eyes were harvested, immediately fixed in 10% neutral buffered formalin for 24 h, then transferred to 70% ethanol, embedded in paraffin. Four-micrometer-thick sections of the eyes from each mouse were cut, processed through graded alcohols. The sections were denitrified by xylene and rehydrated with graded alcohols (100–70%), and stained with hematoxylin and eosin (H&E) (Sigma-Aldrich, St. Louis, MO, United States).

### Immunohistochemical Staining for Neutrophils in the Eyes

For immunohistochemical purposes, after the sections (4-μm) were deparaffinized and rehydrated in distilled water. Heat-induced antigen retrieval was performed in citrate buffer in an 800-W microwave oven for 30 min. Sections were treated with 3% hydrogen peroxide in methanol for 10 min at room temperature to inactivate endogenous peroxidase, and then incubated in 5% normal goat serum in PBS (pH 7.4) for 60 min at room temperature to block non-specific binding. After washing with PBS, sections were incubated with polyclonal anti-elastase (1:100 dilutions) (Boster Biological Technology, Wuhan, China) overnight at 4°C. Negative controls were performed without a primary antibody. Slides were rinsed three times with PBS and were then incubated with the secondary antibody (Goat anti-rabbit IgG) conjugated with horseradish peroxidase for 20 min. The color reaction was revealed by reacting the specimen with a 3,3′-diaminobenzidine substrate (Zhongshan Golden Bridge Technology, Beijing, China). Sections used as isotype controls were incubated with secondary antibodies alone. The sections were counterstained with hematoxylin and positive cells were identified by dark-brown staining under a light microscope.

### Transmission Electron Microscopy

Mice were euthanized at day 8 p.i., and the eyes were dissected, excising the cornea, iris, lens, and vitreous body, and cut into four pieces. The samples were immediately fixed in 3% glutaraldehyde and 1% osmium tetroxide (both in 100 mM PBS, pH 7.2) overnight before being dehydrated through a series of graded ethanol solutions. The fixed tissues were then embedded in SPI-Pon 812 Embedding Kit (Structure Probe Inc., West Chester, PA, United States) following the manufacture’s instruction. Ultrathin sections (70 nm) were cut from the embedded tissues using the Leica EM UC6 ultramicrotome (Leica Microsystems, Wetzlar, Germany) and mounted on formvar-coated grids. The sections were then stained for 15 min in aqueous 1% uranyl acetate followed by 0.2% lead citrate, and were then analyzed under a JEM100CX-II transmission electron microscope (JEOL Ltd., Tokyo, Japan) at an accelerating voltage of 100 kV.

### Statistical Analysis

Statistical analysis was performed using IBM SPSS Statistics version 22.0 (IBM Corp., Armonk, NY, United States). All graphs were generated using GraphPad Prism 7 software (GraphPad software). Data are presented as mean ± standard deviation (SD) at least three independent biological replicates. Student’s *t* test was used to ascertain the differences between groups. Pearson correlation testing was used for the associations between the levels of cytokines. A value of *P* < 0.05 was considered significant.

## Results

### Ocular Pathology and Parasite Burden in the Eyes of B6 and BALB/c Mice Infected With *T. gondii*

**FIGURE 1:**
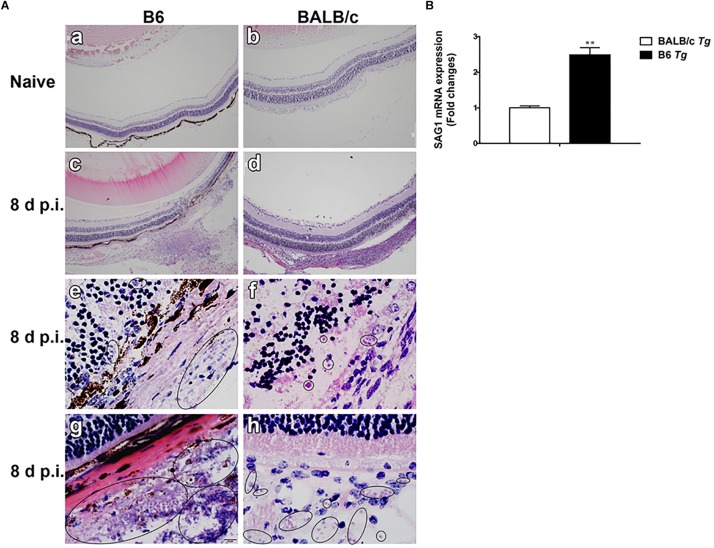
Histological changes **(A)** and parasite burden **(B)** in the eyes of *T. gondii*-infected B6 and BALB/c mice. No histological change was observed in the eyes of uninfected B6 mouse **(a)** and BALB/c mouse **(b)**; at day 8 p.i., severe damage was observed in the eye of *T. gondii*-infected B6 mouse **(c,e,g)** and moderate damage was observed in the eye of *T. gondii*-infected BALB/c mouse **(d,f,h)**. Black circle indicates the tachyzoites of *T. gondii*. The original magnification, **a–d** 100×; **e–h** 1000×, H&E staining. The SAG1 mRNA expressions in the eyes were measured by using qRT-PCR. Data are presented as means ± SD; there were six mice in each group and the data shown are representative of those from two different experiments. *^∗∗^P* < 0.01, *T. gondii*-infected B6 mice vs. *T. gondii*-infected BALB/c mice.

**FIGURE 2:**
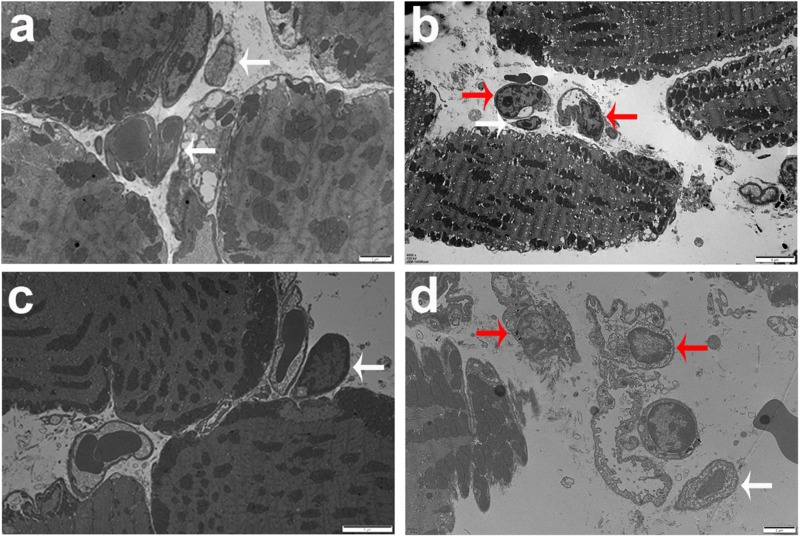
Ultrastructural analysis of eye tissues after *T. gondii* infection. The images of transmission electron microscopy showed *T. gondii* in the eyes of B6 mouse **(a,b)** and BALB/c mouse **(c,d)**. Tachyzoites were indicated by white arrows and neutrophils were indicated by red arrows. Scale bar: a and d were 2 μm; b and c were 5 μm.

Histopathological studies showed that the intravitreal inoculation of tachyzoites of *T. gondii* in both B6 and BALB/c mice caused a strong influx of inflammatory cells, thereby destroying and disrupting the normal structures of retina and choroid as compared with the control groups. In contrast with BALB/c mice, B6 mice showed great multiplication of *T. gondii* tachyzoites with considerable infiltration of numerous migrating cells in the eye tissues at day 8 p.i. (Figure 1A). As for the parasite burden in the eyes with *T. gondii* infection, there was significantly increased mRNA expression level of *T. gondii* tachyzoite SAG1 gene in the eyes of B6 mice than that in BALB/c mice (*P* < 0.01) (Figure 1B). The ultrastructural characterization of *T. gondii* tachyzoites in the eyes of B6 and BALB/c mice were captured by means of transmission electron microscopy. More *T. gondii* tachyzoites were observed in the eyes of B6 mice than those in BALB/c mice (Figure 2).

### Neutrophils in the Eyes of *T. gondii*-Infected B6 and BALB/c Mice

**FIGURE 3:**
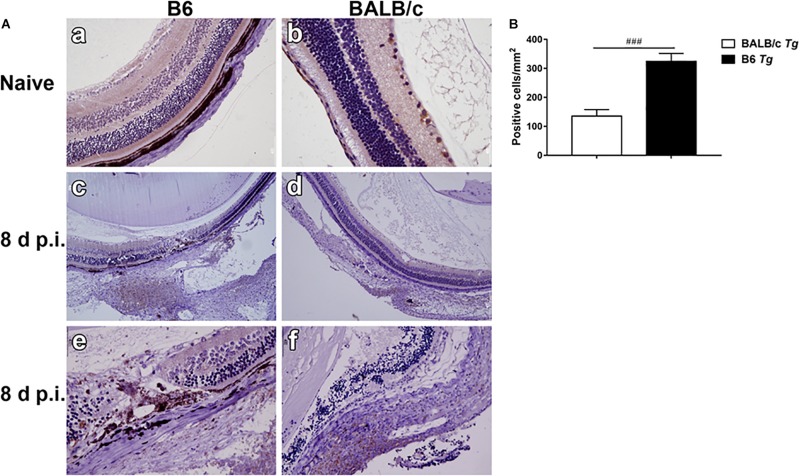
The expression of neutrophil elastase-positive cells in the eyes by immunohistochemical staining **(A)**. Shown are the eyes of an uninfected B6 mouse **(a)** and an uninfected BALB/c mouse **(b)**. At day 8 p.i., there were more neutrophil infiltration in the eyes of *T. gondii*-infected B6 mice **(c,e)** than those in *T. gondii*-infected BALB/c mice **(d,f)**. The original magnification, a and b 200 ×; c and d 100 ×; e and f 400 ×. Quantitative analysis of elastase-positive neutrophil **(B)**. The density of positive cells was expressed as the number of cells per square millimeter. Data are presented as means ± SD; there were six mice in each group and the data represents from two experiments. ^###^*P* < 0.001, *T. gondii*-infected B6 mice vs. *T. gondii*-infected BALB/c mice.

Neutrophils, a kind of inflammatory cell, were observed in the eyes of *T. gondii*-infected mice. Neutrophil elastase is a cytotoxic serine protease, which is stored in the azurophilic granules of neutrophil granulocytes and is released by activated neutrophils. Elastase-positive neutrophils were observed in the destroyed retina and choroid, especially evident in the choroid area of both *T. gondii*-infected B6 and BALB/c mice at day 8 p.i., while they were not observed in those of uninfected B6 and BALB/c mice (Figure 3A). The quantitative analysis showed that compared with *T. gondii-*infected BALB/c mice, the number of neutrophils was significantly higher in the eyes of *T. gondii*-infected B6 mice (*P* < 0.001) (Figure 3B).

### Expressions of TREM-1, TREM-2, IL-1β, IL-33, and ST2 Genes in the Eyes of *T. gondii*-Infected B6 and BALB/c Mice

**FIGURE 4:**
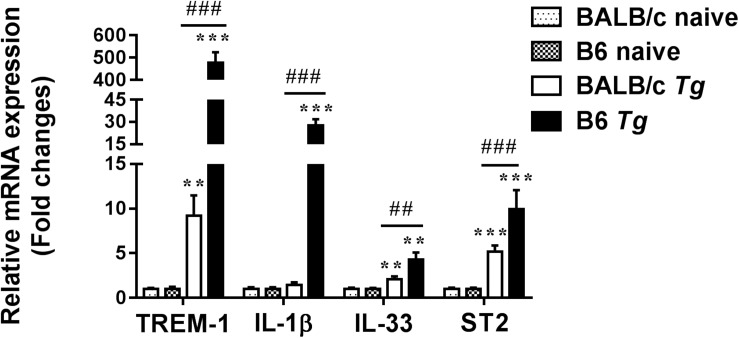
The mRNA expressions of TREM-1, IL-1β, IL-33, ST2, and TREM-2 in the eyes of naive mice and *T. gondii*-infected mice were measured by using qRT-PCR. Data are presented as means ± SD; there were six mice in each group and the data represents from two experiments. *^∗∗^P* < 0.01, and *^∗∗∗^P* < 0.001, *T. gondii*-infected mice vs. naive mice. *^##^P* < 0.01, *^###^P* < 0.001, *T. gondii*-infected B6 mice vs. *T. gondii*-infected BALB/c mice.

Compared with naive mice, the mRNA expression levels of TREM-1 (*P* < 0.01 and *P* < 0.001, respectively), IL-33 (*P* < 0.01), and ST2 (*P* < 0.001) were significantly increased in the eyes of both *T. gondii*-infected B6 and BALB/c mice, while IL-1β expression level was significantly increased in that of *T. gondii*-infected B6 mice at day 8 p.i. (*P* < 0.001). There was insignificant difference in TREM-2 expression levels between *T. gondii*-infected mice and naive mice. Compared with *T. gondii*-infected BALB/c mice, the levels of TREM-1 (*P* < 0.001), IL-1β (*P* < 0.001), IL-33 (*P* < 0.01), and ST2 (*P* < 0.001) were significantly increased in the eyes of *T. gondii*-infected B6 mice (Figure 4).

### Expressions of TLR Genes in the Eyes of *T. gondii*-Infected B6 and BALB/c Mice

**FIGURE 5:**
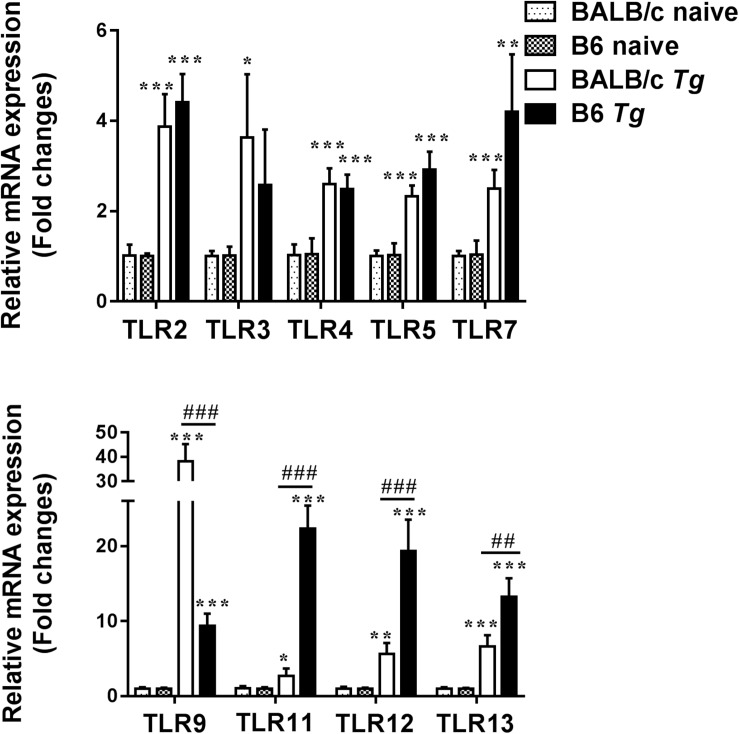
The mRNA expressions of TLRs in the eyes of naive mice and *T. gondii*-infected mice were measured by using qRT-PCR. Data are presented as means ± SD; there were six mice in each group and the data represents from two experiments. *^∗^P* < 0.05, *^∗∗^P* < 0.01, and *^∗∗∗^P* < 0.001, *T. gondii*-infected mice vs. naive mice. ^##^*P* < 0.01 and ^###^*P* < 0.001, *T. gondii*-infected B6 mice vs. *T. gondii*-infected BALB/c mice.

Compared with uninfected control mice, the expression levels of TLR2, TLR4, TLR5, TLR7, TLR9, TLR11, TLR12, and TLR13 in the eyes of both *T. gondii*-infected B6 and BALB/c mice were significantly increased at day 8 p.i. (Figure 5). Compared with *T. gondii*-infected BALB/c mice, the levels of TLR9 (*P* < 0.001), TLR11 (*P* < 0.001), TLR12 (*P* < 0.001), and TLR13 (*P* < 0.01) were significantly increased in the eyes of *T. gondii*-infected B6 mice at day 8 p.i. However, there were significantly elevated levels of TLR3 and TLR9 in the eyes of *T. gondii*-infected BALB/c mice (*P* < 0.05) compared with *T. gondii*-infected B6 mice.

### Correlations Between TREM-1 and IL-1β, IL-33, ST2, or TLRs in the Eyes of *T. gondii*-Infected B6 and BALB/c Mice

**FIGURE 6:**
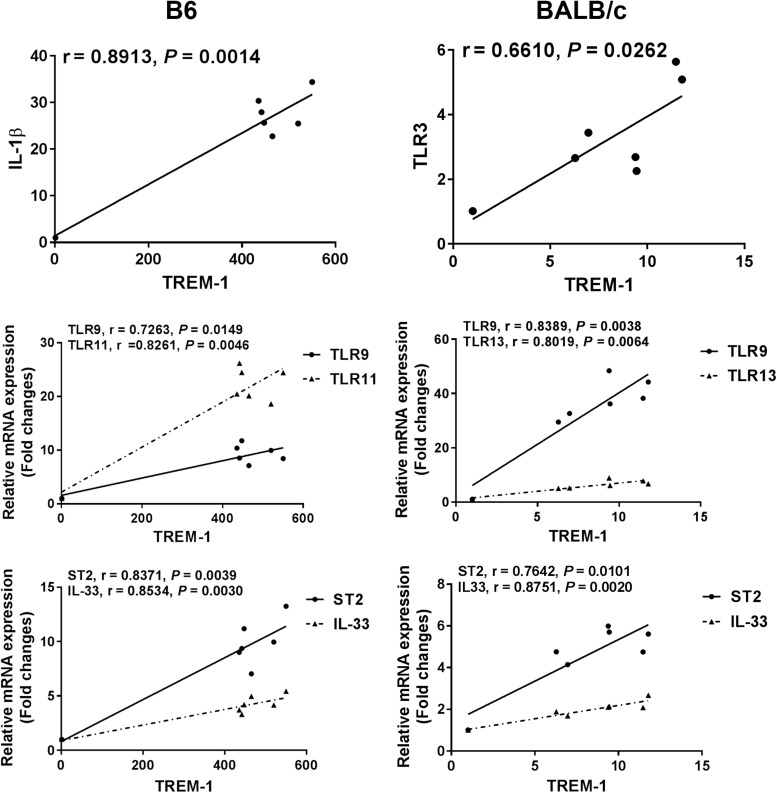
Correlation analysis between the mRNA expressions of TREM-1 and IL-1β, IL-33, ST2, or TLRs in the eyes of *T. gondii*-infected B6 and BALB/c mice. The *r* value generates the theoretical line of best fit, and the *P* value indicates the significance of the correlation. There were six mice in each group and the data represents from two experiments.

The correlations between mRNA levels of TREM-1 and IL-1β/IL-33/ST2/TLRs in the eyes of *T. gondii*–infected B6 and BALB/c mice were analyzed. Only significant correlations were shown. In *T. gondii*-infected B6 mice, there were significant correlations between the mRNA levels of TREM-1 and IL-1β (*r* = 0.8913, *P* = 0.0014), TREM-1 and IL-33 (*r* = 0.8534, *P* = 0.0030), TREM-1 and ST2 (*r* = 0.8371, *P* = 0.0039), TREM-1 and TLR9 (*r* = 0.7263, *P* = 0.0149), and TREM-1 and TLR11 (*r* = 0.8261, *P* = 0.0046). In *T. gondii*-infected BALB/c mice, there were significant correlations between the mRNA levels of TREM-1 and TLR3 (*r* = 0.6610, *P* = 0.0262), TREM-1 and IL-33 (*r* = 0.8751, *P* = 0.0020), TREM-1 and ST2 (*r* = 0.7642, *P* = 0.0101), TREM-1 and TLR9 (*r* = 0.8389, *P* = 0.0038), and TREM-1 and TLR13 (*r* = 0.8019, *P* = 0.0064) (Figure 6).

## Discussion

Our previous study has demonstrated that genetic factors of both mouse strains and parasite strains are crucial in determining susceptibility to experimental murine OT (Lu et al., 2005). IL-10, CD4^+^, CD8^+^ T cells, and B cells play important roles during murine OT (Lu et al., 2003, 2004). Ocular infection caused by *T. gondii* may result in inflammation in the retina, choroid, and uvea (Jasper et al., 2017). In this study, after ocular *T. gondii* infection, there were more severe ocular pathology, more tachyzoites, and higher parasite load in the eyes of B6 mice than those in BALB/c mice. Immunohistochemical staining showed that there was greater neutrophil infiltration in the eyes of B6 mice than those in BALB/c mice following ocular *T. gondii* infection. The differences in expression levels of SAG1 gene and the numbers of neutrophils were consistent with the ocular pathological severity in the two strains of mice. Therefore, our concern is whether the effects of *T. gondii* infection on the expressions of TREM-1, TREM-2, IL-1β, IL-33, ST2, and TLRs are differently in the eyes of *T. gondii*-infected B6 and BALB/c mice.

Triggering receptor expressed on myeloid cells (TREM)-1, a cell surface receptor expressed at high levels on several immune cells such as polymorphonuclear neutrophils, macrophages, and monocytes, plays a vital role in innate and adaptive immune responses (Bouchon et al., 2000). TREM-1 activation increases the release of IL-6, TNFα, and macrophage inflammatory protein-2 as well as polymorphonuclear neutrophil infiltration (Lagler et al., 2009); while TREM-2 may function as a negative regulator in the inflammatory response (Sun et al., 2013). So far, the expression of TREMs is an interesting and as yet unexplored player in OT. In this study, neutrophil infiltration and the expression levels of TREM-1, IL-33, and ST2 increased in the eyes of both B6 and BALB/c mice, and the IL-1β level was increased in B6 mice after ocular *T. gondii* infection, TREM-1 plays a role in regulating neutrophil chemotaxis in acute infectious diseases and is a potential biomarker for the diagnosis of infectious diseases (Cao et al., 2017). TREM-1 alters neutrophil infiltration by stimulating AKT activation and NADPH oxidase-2-dependent superoxide release (Baruah et al., 2019). Blockage of TREM-1 expressed on neutrophils and monocytes/macrophages decreases the activation of neutrophils and monocytes/macrophages and mRNA expressions of inflammation-associated genes in alcoholic liver disease of mouse model (Tornai et al., 2019). IL-1β may play an important role in regulating the host’s immune defense against *T. gondii* infection (Chang et al., 1990). It has been reported that IL-33 is released by damaged or necrotic cells, leading to activation of the immune system by ST2/IL-33 signaling (Moussion et al., 2008; Liew et al., 2010). ST2 increases the Th2 reaction and resistance to *P. aeruginosa* keratitis (Huang et al., 2007). Our previous study found that IL-33/ST2 axis may involve in the regulation of immunopathology of OT in Kunming mice (Tong and Lu, 2015). In the present study, our data demonstrated that both TREM-1 and neutrophil infiltration are essential to *T. gondii*-stimulated ocular inflammatory response, and there were significant positive correlation between the mRNA expressions of TREM-1 and proinflammatory cytokine (IL-1β) in the eyes of B6 mice, and between TREM-1 and IL-33/ST2 in the eyes of both *T. gondii*-infected B6 and BALB/c mice. However, there were significantly higher numbers of neutrophil infiltration and higher levels of TREM-1, IL-1β, and IL-33/ST2 in the eyes of susceptible B6 mice after ocular *T. gondii* infection, which indicate that upregulated TREM-1 may promote inflammation through increased neutrophil recruitment and inflammatory cytokine production in the eyes of susceptible B6 mice.

Multiple TLRs contribute to host innate immunity to *T. gondii* infection, and different TLRs can induce distinctive immune responses to *T. gondii* (Sturge et al., 2013; Han et al., 2014). Our data showed that the expression levels of TLRs (including TLR2, TLR4, TLR5, TLR7, TLR9, TLR11, TLR12, and TLR13) were significantly elevated in the eyes of both B6 and BALB/c mice infected with *T. gondii* through the ocular route. Besides, the levels of TLR11, TLR12, and TLR13 were significantly higher in susceptible B6 mice than those in resistant BALB/c mice, while TLR9 level was significantly higher in resistant BALB/c mice than that in B6 mice. During acute infection, *T. gondii* induces a protective immunity that is mainly Th1 cellular immune response (Denkers and Gazzinelli, 1998). After oral *T. gondii* infection, TLR-9 is crucial for an effective Th1-type immune response in mice (Minns et al., 2006). *T. gondii* profilin recognized by TLR11/12 can induce an inflammatory response, and it can also induce innate and adaptive immune responses (Hedhli et al., 2016). In the current study, we observed that the pathological role of TREM-1 in OT may be associated with the expressions of TLR9/TLR11 in *T. gondii*-infected B6 mice, and associated with the expressions of TLR3/TLR9/TLR13 in *T. gondii*-infected BALB/c mice, suggesting a possibly correlation between TREM-1 and TLR genes during acute OT. It has been reported that after infection with *P. aeruginosa*, TREM-1 mRNA expressions were significantly increased in both human and mouse corneas, which contribute to amplifying corneal inflammation in *P. aeruginosa* keratitis by regulating TLR signaling and immune responses (Wu et al., 2011). Our data suggest that TLR3, TLR9, TLR11, TLR12, and TLR13 may play different roles during ocular *T. gondii* infection.

## Conclusion

This study has provided evidences that following ocular *T. gondii* infection, increased neutrophil infiltration was consistent with the increase of TREM-1 expression in the eyes of both *T. gondii*-infected B6 and BALB/c mice, and significant correlations existed between TREM-1 and IL-1β/TLR9/TLR11/IL-33/ST2 in the eyes of B6 mice and existed between TREM-1 and TLR3/TLR9/TLR13/IL-33/ST2 in the eyes of BALB/c mice, which are possibly related to the ocular immunopathology. However, the functions of TREM-1 and the aforementioned genes related to *T. gondii*-induced immune and inflammatory responses in OT on different background of mice need to be further investigated.

## Data Availability Statement

The raw data supporting the conclusions of this manuscript will be made available by the authors, without undue reservation, to any qualified researcher.

## Ethics Statement

Animal studies were conducted according to protocols approved by the Animal Experimentation Ethics Committee of Zhongshan School of Medicine on Laboratory Animal Care at Sun Yat-sen University (No. 2016-081), China.

## Author Contributions

FL designed the experiments, analyzed the data, wrote, and edited the manuscript. YZ conducted the experiments and analyzed the data. JH and HZ conducted the experiments. SH analyzed the data, revised, and edited the manuscript.

## Conflict of Interest

The authors declare that the research was conducted in the absence of any commercial or financial relationships that could be construed as a potential conflict of interest.
